# Leveraging protein quaternary structure to identify oncogenic driver mutations

**DOI:** 10.1186/s12859-016-0963-3

**Published:** 2016-03-22

**Authors:** Gregory A. Ryslik, Yuwei Cheng, Yorgo Modis, Hongyu Zhao

**Affiliations:** Department of Biostatistics, Yale School of Public Health, New Haven, CT USA; Program of Computational Biology and Bioinformatics, Yale University, New Haven, CT USA; Department of Medicine, University of Cambridge, MRC Laboratory of Molecular Biology, Francis Crick Avenue, Cambridge, CB2 0QH UK

## Abstract

**Background:**

Identifying key “driver” mutations which are responsible for tumorigenesis is critical in the development of new oncology drugs. Due to multiple pharmacological successes in treating cancers that are caused by such driver mutations, a large body of methods have been developed to differentiate these mutations from the benign “passenger” mutations which occur in the tumor but do not further progress the disease. Under the hypothesis that driver mutations tend to cluster in key regions of the protein, the development of algorithms that identify these clusters has become a critical area of research.

**Results:**

We have developed a novel methodology, *QuartPAC* (**Q**uaternary **P**rotein **A**mino acid **C**lustering), that identifies non-random mutational clustering while utilizing the protein quaternary structure in 3D space. By integrating the spatial information in the Protein Data Bank (PDB) and the mutational data in the Catalogue of Somatic Mutations in Cancer (COSMIC), *QuartPAC* is able to identify clusters which are otherwise missed in a variety of proteins. The R package is available on Bioconductor at: http://bioconductor.jp/packages/3.1/bioc/html/QuartPAC.html.

**Conclusion:**

*QuartPAC* provides a unique tool to identify mutational clustering while accounting for the complete folded protein quaternary structure.

**Electronic supplementary material:**

The online version of this article (doi:10.1186/s12859-016-0963-3) contains supplementary material, which is available to authorized users.

## Background

Cancer, one of the most costly and heterogenous diseases, is ultimately caused by a build up of somatic mutations within oncogenes or tumor suppressors [1]. Typically, oncogenic mutations result in an increase of gene output or a destabilization of the the resulting protein while mutations within tumor suppressors lead to a reduction of gene activities that promote apoptosis or cell cycle regulation. Due to the relative ease of disrupting protein function as compared to restoring it, significant pharmacological progress has been made towards inhibiting oncogenic mutations as shown by [2–4]. Combined with the theory of oncogene addiction, that a small subset of so called driver genes result in runaway cellular replication and that the selective targeting of these genes can have a large impact on tumorigenesis [5, 6], the identification of such driver genes becomes critical due to the large translational benefit in the pharmacological space.

Due to the medicinal and biological importance of identifying these driver mutations, a large ensemble of methodologies have been developed. One popular approach is based on the hypothesis that driver mutations have a higher frequency of non-synonymous mutations when compared to the background mutation rate [7, 8]. Relatedly, several studies have shown that somatic mutations cluster within protein kinases [6, 8–10] and that these clusters may be a sign of positive selection for protein function and thus targets for therapeutic intervention [11, 12]. Such frequency based approaches at identifying driver mutations are often further augmented by accounting for a variety factors such as normalizing for gene length [13], accounting for tumor type and varying background mutation rates [13, 14], as well as considering the ratio of nonsynonymous (*K*_*a*_) to synonymous (*K*_*s*_) mutations [15].

In addition to the above methods, several machine learners have been designed to determine the impact of a specific mutation. For example, *CHASM* [16] endeavors to classify between driver and passenger mutations while *Polyphen-2* [17] attempts to determine if a mutation is damaging or benign. Overall, the machine learning approaches utilize a large set of “features” such as sequence, size and polarity of the substituted residues, as well as whether the mutation occurred in a conserved region [18]. These features are used to build a set of rules which are then used to score each mutation. The value of the score then determines how detrimental is the mutation or is used to classify the mutation into a particular category, for example “driver” versus “passenger”. While some classifiers are designed to handle a large feature space, others are optimized to use only a subset of these features. For instance, *SIFT* only considers the degree of evolutionary conservation when determining whether an amino acid substitution affects protein function [19]. Once the feature set has been determined, a variety of statistical learners such as Random Forests [20], Support Vector Machines [21] and Bayesian Networks [22] are then used to build the model.

Although all of the above methods have shown success in determining whether a mutation is harmful, they nevertheless have limitations as well. Machine learners for example often require several sources of information that must be periodically updated, often at significant expense. Approaches that rely upon differentiating between the frequency of *K*_*a*_ to *K*_*s*_ over the entire gene may fail if selection only occurs upon a small region of the gene. Similarly, approaches such as those proposed by [14] lose accuracy if the background mutation rate can not be precisely calculated. Other algorithms, such as those proposed by [13, 15] do not distinguish between activating and non-activating mutations.

Using the hypothesis that activating mutations cluster in functionally significant protein regions, [23–26] have developed several approaches to identify mutational clustering. Ye et al. [23] created Non-Random Mutational Clustering (*NMC*) by testing against the null hypothesis that non-synonymous amino acid mutations are distributed uniformly along the polypeptide. However, the algorithm is based upon order statistics and thus considers the protein as a linear sequence of amino acids without taking protein structure into account. To that end, *iPAC* [24] and *GraphPAC* [25] extended *NMC* to account for protein tertiary structure. While both approaches remapped the protein to one dimensional space before identifying clustering, *iPAC* utilized a global remapping via Multidimensional Scaling (MDS) while *GraphPAC* employed a local remapping via a graph theoretical approach. While both of these methods considered the protein tertiary structure when identifying clustering, they nevertheless required a remapping to one dimension which resulted in information loss. As such, *SpacePAC* [26] performed a simulation based analysis to identify clustering directly in 3D space. Despite the success of the above methods, they nevertheless only consider up to the protein tertiary structure and do not account for the large complexes that the protein subunits create *in vivo* when performing biological functions.

In this article, we extend the work done by *iPAC*, *GraphPAC* and *SpacePAC* to consider protein quaternary structure when identifying mutational clusters. This approach allows us to detect clusters that become apparent only when there are multiple polypeptide chains in the complex. For example, statistically significant clusters in structures *1SUV*, *2GRN* and *2YDR* are identified only when the entire protein complex is considered (see ‘Sections *iPAC* identifies new proteins with clustering’, ‘*GraphPAC* identifies new proteins with clustering’, and ‘*SpacePAC* identifies new proteins with clustering’). Furthermore, *QuartPAC* detects additional mutational hotspots in proteins known to have clustering and thus expands the repertoire of pharmacological targets that can be investigated. We also evaluate the performance of *QuartPAC* when identifying mutations that are classified as damaging or driver mutations by *PolyPhen-2* and *CHASM*, respectively. In all, by accounting for the highest level of protein complexity, we are able to discern clusters that are otherwise missed by algorithms that only consider the protein tertiary structure.

## Methods

The *QuartPAC* methodology consists of three main parts. The first part obtains the mutational and structural data for each subunit in the quaternary complex (see Section ‘Obtaining mutational & structural data’). The next step is to reconcile the quaternary protein structural information with the mutational data so that the correct mutation is mapped onto the proper amino acid (see Section ‘Reconciling structural and mutational data’). The final step is to run the underlying clustering algorithm on the reconciled quaternary structure (Section ‘Identifying mutational clusters’). For this manuscript, we executed the algorithms presented in *iPAC*, *GraphPAC* and *SpacePAC* in order to identify statistically significant clusters. The software allows the user to specify which clustering algorithms they want to utilize. Lastly, although not part of the *QuartPAC* process, we correct for the multiple comparison penalty as we test many structures for clustering (see Section ‘Multiple comparison adjustment for structures’). We also note that we use the term “cluster” and “hotspot” interchangeably throughout this manuscript.

### Obtaining mutational & structural data

The 70th version of the COSMIC database, the most recent as of when this article was drafted (available via http://cancer.sanger.ac.uk/cosmic), was used to retrieve the mutational data. In order for us to include a mutation in our analysis, it first needed to meet several criteria. First, only nonsynonymous missense mutations that were classified as a “confirmed somatic variant” or “Reported in another sample as somatic” were retained. Next, as all the clustering algorithms test against the null hypothesis that mutations are randomly and uniformly distributed along the polypeptide chain, in order to avoid selection bias, only mutations from whole genome or whole gene screens were kept. Further, as multiple studies often report or use the same mutational data from a single cell line, all the mutations were screened in order to remove duplicate mutations and avoid double counting specific variants. Finally, the gene on which the mutation occurred must of been properly labeled with a Uniprot Accession Number [27]. This allowed us to correctly match the mutation to the protein structure in the PDB (see “COSMIC Query.docx” in Additional file 1 for the entire SQL query).

The structural information was accessed from the PDB by cross-referencing the uniprots from the COSMIC database against those for which quaternary structural information was available. Since multiple structures are often available for the same protein subunits (or a subset of the same subunits), all relevant structures with matching Uniprot Accession Numbers were kept and a multiple comparison adjustment applied afterwards (see Section ‘Multiple comparison adjustment for structures’). In addition, as every amino acid is comprised of several atoms, the (x,y,z) coordinates of the *α*-carbon atom were used to represent amino acid positions. As shown in [25], using other backbone atoms such as the amide nitrogen or main chain carbonyl carbon is possible but has minimal effect. For a full listing of the 2267 structures considered for analysis, see Additional file 2: Structure files.xlsx in Supplementary materials.

We note that while each PDB entry was used once and only once in each analysis, proteins present in multiple PDB entries are analyzed multiple times. As a given protein can adopt different structures due to a variety of factors, such as variations in the amino acid sequence or the presence of other bound proteins or cofactors, it is important to consider all possible structures. Indeed, one specific structure may be the one that provides insight into the oncogenic process while the other structures do not. However, should only one structure per protein be considered our results would be even more significant as the multiple comparison penalty (see Section ‘Multiple comparison adjustment for structures’) would be reduced.

### Reconciling structural and mutational data

As the residue numbering in the PDB database does not match the canonical residue numbering in the COSMIC database, a reconciliation is required in order to map the mutational data to the structural data. Similar to *iPAC*, *GraphPAC* and *SpacePAC*, a pairwise alignment was performed as detailed in [28]. Should the user so desire, a manual alignment is also possible. For full details on the pairwise alignment algorithm, consult the *iPAC* package available on Bioconductor (http://www.bioconductor.org/packages/release/bioc/html/iPAC.html). Successful alignment was obtained on 2156 quaternary protein structures for which applicable uniprot information was available. Structures for which there were fewer than two mutations were labeled as blank (since no clustering was possible). Refer to “Methodology Results.xlsx” in Additional file 3 for a full listing of the 2156 structures that had a successful alignment and were statistically analyzed.

### Identifying mutational clusters

The underlying approach for *QuartPAC* is that it performs each of the clustering approaches specified in *iPAC*, *GraphPAC* and *SpacePAC* but on the quaternary protein structure. As such, the complexity of the methodology presented here stems from correctly handling the folded structure of the protein subunits when they come together to form a macromolecule. We describe briefly each of the clustering methodologies below and refer the reader to the original manuscripts for further details.

#### *iPAC*

The *iPAC* methodology remaps the protein from $\mathbb {R}^{3} \rightarrow \mathbb {R}$ by minimizing the stress function defined as:

(1) $$\begin{array}{@{}rcl@{}} \sigma_{1} = \sqrt{\frac{\sum_{i,j}\left[f(\delta_{i,j}) - d_{i,j}(\mathbf{X})\right]^{2}}{\sum_{i,j}d_{i,j}^{2}(\mathbf{X})}} \end{array} $$

In the equation above, *δ*_*i*,*j*_ represents the distance between the *α*-carbon atoms of residues *i* and *j* in $\mathbb {R}^{3}$ and *d*_*i*,*j*_(*X*) represents the distance between the residues in the lower dimensional space *X*. In our case, *X* is the line, $\mathbb {R}$. Finally, *f* is used when the original space is not a metric space. Since the protein is in $\mathbb {R}^{3}$, we simply have *f* to be the identity function. The denominator of the expression is used to ensure that the remapping is the same regardless of the units used to measure distance.

**Fig. 1:**
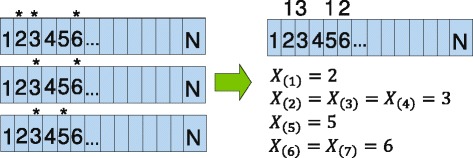
Order statistic construction. Suppose there are a total of seven mutations over three samples of the same protein. The protein is N amino acids long and the number in each box represents the amino acid position. A star above the box signifies a non-synonymous mutation. *X*_(*i*)_=*j* then signifies that the i-th mutation occurred on residue j [24]

By performing a global minimization of *σ*_1_, all pairwise $\mathbb {R}^{3}$ distances are preserved, as best as possible, when the protein is mapped to $\mathbb {R}$. Once in the lower dimensional space, the position of every mutation is utilized to build order statistics as shown in Fig. 1.

Once the order statistics are calculated, a cluster is found between two mutations if *Pr*(*X*_(*k*)_−*X*_(*i*)_)≤*α* for a significance level *α* where *X*_(*i*)_,*X*_(*k*)_ represent the i-th and k-th mutations, respectively, along the reordered amino acid sequence. Typically, *α* is set to be 5 *%* (as is the case for this manuscript as well as for [24–26]), but can be set to whatever level of statistical significance is desired by the study authors. This probability is then calculated for all pairwise mutations and an appropriate multiple comparison adjustment is applied. For the purposes of this paper, a conservative Bonferroni multiple comparisons method was applied to account for all intra-protein comparisons.

#### *GraphPAC*

**Fig. 2:**
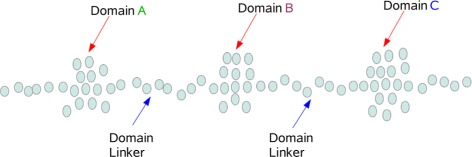
In this cartoon protein, the residues in domain A will be visited by the traveling salesman before any of the residues in Domain B or C. Thus the residues in domains B and C do not impact the remapping of domain A to $\mathbb {R}$. Under *iPAC* however, every amino acid affects every other amino acid’s final position [25]

*GraphPAC* functions similarly to *iPAC* in that it also hinges on a mapping from $\mathbb {R}^{3} \rightarrow \mathbb {R}$. However, *GraphPAC* performs a local minimization by only considering nearby residues when projecting down onto the lower dimensional space. For instance, as shown in Fig. 2, the *iPAC* methodology will allow for residues in Domain C to have an effect on the final position of residues in Domain A and vice versa. However, utilizing the *GraphPAC* approach, only nearby residues will effect the remapping process.

To achieve this “local-based” reordering, *GraphPAC* utilizes a graph theoretic algorithm. Specifically, the algorithm sets every residue to be a vertex and all vertices are then connected to one another forming a complete graph. The weight on the edge between vertices i and j is set to be equal to the Euclidean distance between amino acids i and j in $\mathbb {R}^{3}$. A heuristic approach is then used to solve the traveling salesman problem in order to find the shortest Hamiltonian path through the protein. In particular, we attempt to heuristically identify the permutation *π* that solves:

(2) $$ \min_{\pi} \sum\limits_{i=1}^{n} d(i, \pi(i))  $$

where *π*(*i*) represents the amino acid that follows residue *i* on a path through the protein. While there are many heuristic solutions to the TSP, the problem is NP-hard and there is no known solution that can be solved in polynomial time. However, as shown by [25], the results are remarkably consistent no matter what heuristic approach is used.

#### *SpacePAC*

Unlike *iPAC* and *GraphPAC*, *SpacePAC* attempts to identify clustering directly in $\mathbb {R}^{3}$ by identifying the one, two and three non-overlapping spheres that cover the greatest number of mutations possible at different sphere radii lengths. This statistic is then compared to simulated values in order to come up with a *p*-value. As described in [26], the specific procedure is:

- Let s be the number of spheres we consider; *s*∈{1,2,3}.
- Let r be the radius considered. Here we consider, *r*∈{1,2,3,4,5,6,7,8,9,10} Ångstroms.
- Simulate *T*(≥1000) distributions of mutation locations over the protein structure. Specifically, for each simulation, every mutation is randomly assigned to a residue *i* where 1≤*i*≤*N* and *N* is the total number of residues in the protein quaternary structure.

Next, let *X*_*i,s,r*_ represent the number of mutations captured in simulation *i* (where *i*=0 represents the observed data), *s*∈{1,2,3} represents the number of spheres used and *r* represents the radius of each sphere. Then for a given {*s*,*r*} combination,

(3) $$ \mu_{s,r} = \underset{1 \leq i \leq T}{\text{mean}}\{X_{\text{\textit{i,s,r}}}\},  $$

(4) $$ \sigma_{s,r} = \underset{1 \leq i \leq T}{\text{std.\ dev.}}\{X_{\text{\textit{i,s,r}}}\}  $$

(5) $$ Z_{i} = \max_{s,r} \{ (X_{\text{\textit{i,s,r}}}- \mu_{s,r})/\sigma_{s,r} \}  $$

**Fig. 3:**
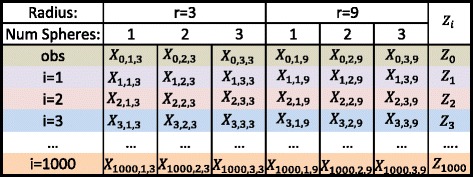
Statistic construction. Here we consider up to three spheres (*s*∈{1,2,3}) and radii of either 3 or 9 Å. The first step is to calculate *μ* and *σ* over each column and then a normalized statistic $Z_{{i,s,r}} = \frac {X_{i,s,r }- \mu _{s,r}}{\sigma _{s,r}}$ for each cell. Then the maximum is taken over each row, specifically *m* *a* *x*_*s*,*r*_ *Z*_*i,s,r*_, to obtain *Z*_0_,…,*Z*_1000_. One minus the percentage of cases where *Z*_0_≥*Z*_*i*_, for *i*∈{1,…,1000}, is the *p*-value of our observed statistic *Z*_0_. As 1000 simulations were run, if *Z*_0_>*Z*_*i*_ ∀*i*, a *p*-value <1.00*E*−03 is reported [26]

Once the normalized statistics *Z*_*i*_ are calculated, the *p*-value is estimated as $1 - \left (\sum {\mathbf {1}_{Z_{0} > Z_{i}}}\right) / T$. Thus per every run of the simulation, there is only one *p*-value necessary to identify the statistical significance of up to *s* hot spots. A visual layout of the calculation of this statistic is shown in Fig. 3. It is also worth noting that given *n* positions and *m* spheres, there are $n \choose m$ sphere orientations possible that must be checked under a brute force approach. See [26] for a more efficient approach, which is utilized in the analysis for this manuscript, that nevertheless identifies the globally optimum solution.

### Multiple comparison adjustment for structures

A multiple comparison adjustment was made to account for considering the 2156 successfully aligned protein quaternary structures. As multiple structures may be comprised of the same protein subunits, a Bonferroni adjustment was too conservative and an FDR approach was performed. Namely, a rough FDR (rFDR) [29] approach, which approximates the standard FDR methodology [30], was employed due to the large number of potentially positively correlated tests. For this paper, the cutoff was:

(6) $$ rFDR = \alpha\left(\frac{k+1}{2k}\right)  $$

where *k*=2156, the total number of structures in the study. Using an *α*=0.05, the *rFDR*≈0.025012. To be conservative, we rounded down and deemed all clusters with a *p*-value less than or equal to 0.025 to be significant. Further, for the rest of this manuscript we may refer to *iPAC* and *GraphPAC* as the “pairwise” approaches as they require a multiple comparison adjustment for each pair of mutations while *SpacePAC* does not.

## Results and discussion

**Fig. 4:**
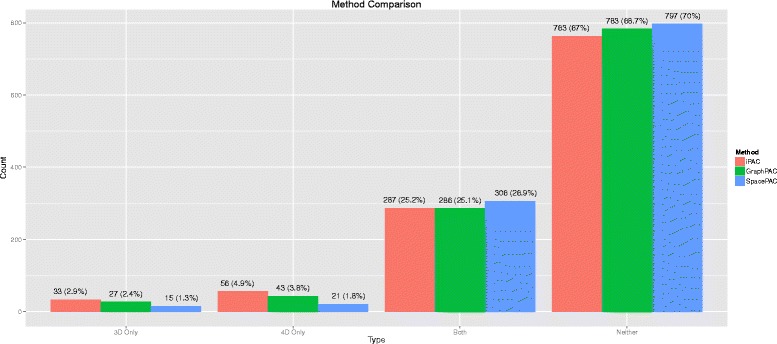
A cross-classification of the structures that were successfully aligned by each method after removing blanks. The colors represent the algorithm used to identify clustering: *iPAC*, *GraphPAC* or *SpacePAC*. An incremental count for “3D only” or “4D only” represents that at least one statistically significant cluster was found only when the tertiary or quaternary structure was considered, respectively. An incremental count for “Both” or “Neither” signifies that the results were concordant regardless of whether the tertiary or quaternary structure was considered

Of the 2156 structures considered, if blanks are removed^1^, approximately 1–5 % of the structures are identified to have clustering only when the protein quaternary structure is considered. Furthermore, approximately 1–3 % of the structures are identified to have clustering only when the protein tertiary structure is considered. For the vast majority of structures, both the tertiary and quaternary algorithms are concordant in whether they identify at least one statistically significant cluster in the structure. The results of each algorithm cross-classified by tertiary versus quaternary classification are shown in Fig. 4 below.

For structures that were identified under only the tertiary methodologies, it is likely that the significant clusters were close to the adjusted *p*-value threshold and when the entire protein complex was considered the additional multiple comparison penalty was high enough to negate the statistical significance. As such, if a quaternary structure is available, it would be statistically preferable to use in order to reduce potential false positives. For a detailed comparison of which structures were identified by the tertiary and quaternary methods, see “Quaternary vs Tertiary.xlsx” in Additional file 4.

**Fig. 5:**
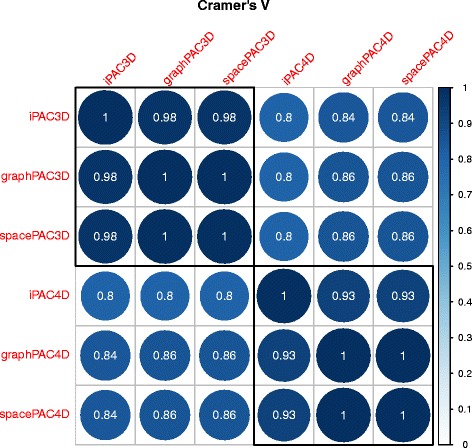
A correlation plot between each of the six methods. A hierarchical clustering approach was applied to group the methods into two categories. As can be seen, the methods that rely upon the tertiary structure separate out from the methods that rely upon the quaternary structure. The number inside the circle is the value for Cramer’s V between the two methods. We note that all the values are statistically significant at the 5 % *α*-level

In Fig. 5, we consider the correlation between each of these methods on a per structure basis. Because cluster counts are not directly comparable between *SpacePAC* and the other two approaches, we applied a nominal classification of three categories: 1) clustering detected, 2) no clustering detected and 3) blank. Cramer’s V [31], was then used to calculate the correlation coefficient between each approach. For reference, Cramer’s V $ = \sqrt {\frac {\chi ^{2}/n}{min(k-1, r-1)}}$ where *χ*^2^ is the statistic from Pearson’s Chi-Squared Test, *k* is the number of columns, *r* is the number of rows, and *n* is the grand total number of observations of pairs (*A*_*i*_,*B*_*i*_). Here, *A*_*i*_=1 represents whether structure *i* had a statistically significant cluster under method A (otherwise *A*_*i*_=0) and *B*_*i*_ represents whether structure *i* had a statistically significant cluster under method B (otherwise *B*_*i*_=0). For the purposes of this manuscript, as we are comparing all six pairwise methods over the 2156 structures, *k*=2 and *r*=2156 for every pairwise-algorithmic comparison.

**Fig. 6:**
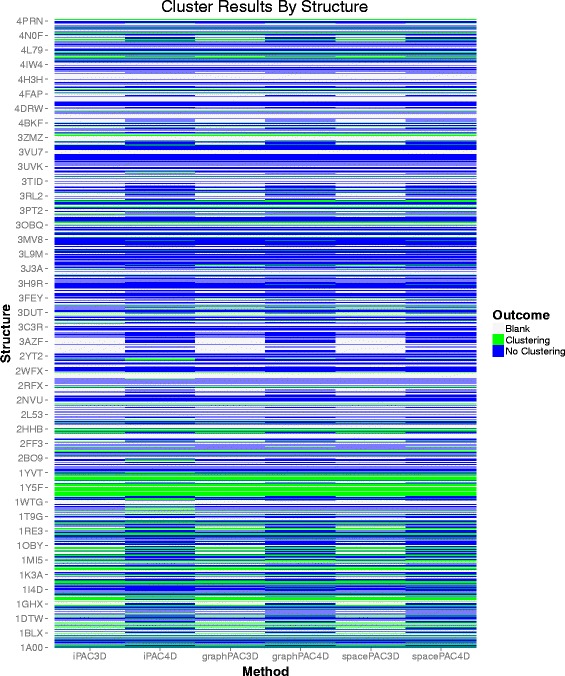
A modified heat map that shows whether the structure contained statistically significant clusters or not. Structures with fewer than two mutations were considered as blanks

Figure 6 below presents a per structure view comparison between the two methods when the structures are considered in decreasing lexicographic order. We believe that a hierarchical reordering of the structures is not appropriate in this case due to the fact that we once again consider only the trinary outcome of “clustering”, “no clustering” and “blank”. However, from Fig. 6, it is clear that for many structures, what is considered a “blank” becomes a result with “no clustering” when the larger quaternary structure is considered. This is due to the case that when all the subunits in the quaternary structure are considered, it is more likely to observe at least two mutations. As such, the structure is no longer considered to be blank and whether there is clustering or not can now be determined. As can be seen from Fig. 6, this pattern of “blanks” being converted to “no clustering” is consistent for all three methods: *iPAC*, *GraphPAC* and *SpacePAC*. Please see Additional file 5 “Trinary Outcomes.xlsx” for the specific details for each structure.

Table 1 shows the top five statistically significant structures found by each of the spatial methods when considering quaternary structure. As can be seen from the table, while there is significant overlap, there are differences between the algorithms in regards to which structures are identified. This is analogous to when the tertiary structure is considered and suggests that while one should look at the quaternary structure as opposed to the tertiary structure, looking at the macromolecule does not make one of the spatial approaches perform significantly better. Refer to “Methodology Results.xlsx” in the Additional file 3 for a full listing of all 2156 structures along with the clustering results when tertiary and quaternary structures are considered. While it is outside the scope of this paper to go through every protein structure identified to have clustering individually, we note that many of the complexes that we identify when we consider quaternary structure have biological implications. For example, structure *2YDR* contains the TP53 subunit, one of the most common tumor suppressors that has been implicated in a large variety of human cancers [32–34]. Alternatively, structure *4MNQ* from Table 1 contains the HLA class I histocompatibility antigen which plays a significant functional role in the immune system and has recently been associated with lung cancer [35]. In Sections *iPAC* identifies new proteins with clustering, *GraphPAC* identifies new proteins with clustering and *SpacePAC* identifies new proteins with clustering, we cover three representative structures in further detail.

**Table 1:** Summary of the top five most statistically significant structures for each method when using the quaternary structure

	*P*-value		
Structure	iPAC	GraphPAC	SpacePAC
2YDR	1.89E-13	6.27E-18	<0.001
1SUV	2.39E-06	1.57E-05	
3W14	4.57E-06		
1DTW	5.22E-06		
1U5B	2.22E-05		
3V8X		1.49E-05	
4MNQ		1.48E-04	
1QVO		1.60E-04	<0.001
1I5K			<0.001
3B13			<0.001
1A9W			0.002

A blank entry in position (*i*,*j*) denotes that methodology *j* did not find that structure to be statistically significant. We note that if a structure had *n* total mutations, then the pairwise methodologies of *iPAC* and *GraphPAC* calculate $\frac {n(n-1)}{2}$ comparisons, one for each pair of mutations. Therefore, the *p*-values shown for *iPAC* and *GraphPAC* are shown post a Bonferroni correction. For *SpacePAC*, as 1000 simulations were run for each structure, the minimum possible *p*-value we can report is *p*<1.00*E*−03. Please see [26] for more details

Next, we considered the performance by *iPAC*, *GraphPAC* and *SpacePAC* when the quaternary structure is utilized as compared to *PolyPhen-2* [17] and *CHASM* [16]. Both *PolyPhen-2* and *CHASM* utilize a large set of features when evaluating each mutation while *QuartPAC* runs with vastly less a priori information. We note that in order to do a fair comparison, while the quaternary methodologies evaluated each structure, the machine learners evaluated all the protein subunits in each structure. Thus, if at least one subunit had a significant finding under the machine learning methodology, we counted it as a significant finding for the entire quaternary structure. Out of the 343 significant structures found by *iPAC* to contain mutational clustering when considering quaternary structure, *PolyPhen-2* identifies 145 (42 %) structures as having damaging mutations while CHASM identifies 78 (23 %) structures containing driver mutations when using the standard FDR of 20 %. While *GraphPAC* identified 329 structures with significant clustering, *PolyPhen-2* identified 131 (40 %) structures with potentially damaging mutations while *CHASM* identified 89 (27 %) structures. Of the 327 structures identified by SpacePAC as significant, 129 (40 %) and 74 (23 %) structures were identified by *PolyPhen-2* and *CHASM* respectively. These results are summarized in Table 2 below.

**Table 2:** This table summarizes how many of the structures identified as having a significant cluster by one of the quaternary methodologies also had a subunit that had at least one damaging (in the case of *Polyphen-2*) or driver (in the case of *CHASM*) mutation

	Polyphen-2			CHASM	Total flagged
Method	Benign	Possibly damaging	Probably damaging	FDR ≤ 0.2	by quaternary approach
(1)	(2)	(3)	(4)	(5)	(6)
iPAC	198	11	134	78	343
GraphPAC	198	11	120	89	329
SpacePAC	198	11	118	74	327

Column (1) specifies the quaternary methodology used and column (6) denotes how many total structures were flagged using that quaternary approach. Columns (3)-(5) break out the results by the specific machine learning approach

We note, that in [24–26] the overlap between the machine learning approaches and the tertiary methodologies was larger. As the machine learners do not account for the other subunits in the folded protein structure, they flag fewer proteins as having damaging mutations due to the fact they do not leverage the information from the entire folded protein structure, but rather from one protein subunit. As such, the quaternary methodology may increase the chances of finding a critical mutational area when used in conjunction with other machine learning algorithms. See “Performance Evaluation.xlsx” in Additional file 6 for a breakout per structure.

Finally, we compared our results to the data in the OMIM (Online Mendelian Inheritance in Man) [36]. To do this, we cross-tabulated all the 2156 structures we considered and identified their matching entries on a per-gene level in the OMIM database. Each of these genes in the OMIM database was then classified as a binary “true” or “false” where “true” signifies that the gene was denoted to be either causal or related to a disease. This pairing was completed using the most up-to-date version of the OMIM database available as of January 16th, 2016. The results of this analysis, when considering structures found only by tertiary or quaternary methods, are shown in Table 3 below and further details are available in “OMIM classification.xlsx” in Additional file 7.

**Table 3:** The *p*-value represents the results of a one-sided binomial hypothesis test where *H*_0_:*p*_0_=*p*_1_ and *H*_*a*_:*p*_0_>*p*_1_ where *p*_0_ is the proportion of structures found that had a corresponding entry in OMIM when using the quaternary version of the method and *p*_1_ is the proportion of structures with a corresponding entry in OMIM when using the tertiary version of the method

	Quaternary only		Tertiary only		*p*-value
Method	Num structures	Num in OMIM	Num structures	Num in OMIM	
iPAC	56	42 (75 %)	33	8 (24 %)	4.49×10^−6^
GraphPAC	43	31 (72 %)	27	10 (37 %)	4.04×10^−3^
SpacePAC	21	11 (52 %)	15	5 (33 %)	0.214

As can be seen from Table 3 there were significantly more structures found by the quaternary versions of *iPAC* and *GraphPAC* with related OMIM entries. While the difference was not statistically significant for *SpacePAC*, that was mainly due to the fact that *SpacePAC* had much less of a discrepancy between structures that were found only under quaternary and only under tertiary approaches. An expanded version of this table, which considers structures found by both tertiary and quaternary methods combined, is available in Additional file 7 “OMIM classification.xlsx” file. Further, we would like to mention two important observations when analyzing our results in comparison with the OMIM data. First, it is important to note that the OMIM database is not all-inclusive; namely there could very well be genes with hotspots that are oncogenic but which have not been added to the database as of yet. Second, the quaternary methodology described in this manuscript is meant to provide the wet-bench researcher with additional statistically significant clusters. While these clusters may be potential therapeutic targets, final confirmation lies further downstream in the development process and is beyond the scope of this text.

### *iPAC* identifies new proteins with clustering

Under *iPAC*, there were 56 structures that were identified only when considering the protein quaternary structure. While it is outside the scope of this manuscript to go through each one in detail, we present an example from this set. Specifically, we will now consider *1SUV* [37], the structure of human transferrin receptor-transferrin complex. This structure is composed of Transferrin Receptor Protein 1 (TFR1) as well as the C-lobe and N-lobe of serotransferrin. Transferrin proteins, which control the level of free iron, are plasma glycoproteins which are encoded by the TF gene [38, 39]. Recently, it was shown that elevated expression of TFR1 contributes to the oncogenic signaling performed by Sphingosine Kinase 1 (SK1), which in elevated levels enhances cell survival, proliferation and can induce neoplastic transformation. Moreover, by blocking TFR1 with a neutralizing antibody, SK1-induced abnormal cell growth is inhibited which suggests that TFR1 presents a potential therapeutic target for SK1-mediated tumorigenesis [40].

The statistically significant clusters are shown in Table 4 with the clusters referenced by their serial number within the structure file. We note that in addition to the oncogenic implications described above, cluster III also contains mutation G277S in the serotransferrin protein (Uniprot ID: P02787) which is associated with a reduction in total iron binding capacity and is a risk factor for iron deficiency anemia [41].

**Table 4:** Clusters identified by *iPAC* for structure *1SUV*

Cluster	Residues in cluster	Start serial	End serial	Num. Muts	*p*-value
1	233	12570	14345	7	2.39E-06
2	165	13099	14345	4	1.96E-05
3	98	14345	15138	2	3.44E-05
4	98	11777	12570	2	3.50E-05
5	295	10288	12570	6	1.35E-04
6	494	10531	14345	11	1.77E-04
7	166	11777	13099	5	1.80E-04
8	371	10965	13855	9	2.73E-04
9	438	10965	14345	10	2.97E-04
10	427	11777	15138	9	3.17E-04
11	98	13099	13855	3	3.79E-04

For each cluster we show: 1) the number of residues in the cluster, 2) the beginning and ending serial number, 3) the number of mutations in the cluster and 4) the *p*-value

**Fig. 7:**
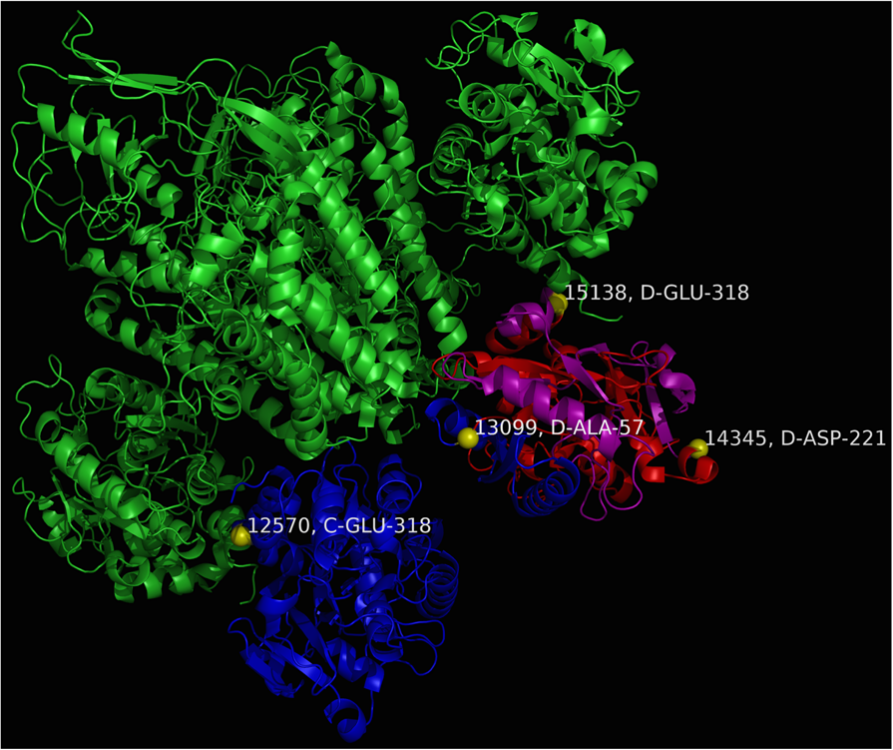
The residues at serial numbers 12570, 13099, 14345 and 15138 are labeled and highlighted in yellow. As cluster II is a subset of cluster I, we have colored the atoms between 12570 and 13099 blue, the atoms between 13099 and 14345 red and the atoms between 14345 and 15138 purple. The rest of the structure is colored in green

The structure of *1SUV* is shown below in Fig. 7 below with the boundaries displayed in Table 4 colored in yellow.

We note that had the entire structure not been considered, no significant clusters are found, signifying that the biological quaternary unit resulted in more mutations within close proximity than any one tertiary substructure alone.

### *GraphPAC* identifies new proteins with clustering

We now proceed to consider structure *2GRN* [42], one of the 43 structures found to be significant by *GraphPAC* only when the quaternary structure is considered. *2GRN* is comprised of two molecules, Ubiquitin-conjugating enzyme E2I which is coded by UBE2I and Ran GTPase-activating Protein 1 which is coded by RANGAP1. Protein ubiquitination is a critical post-translational modification where ubiquitin is added to a substrate protein. This in turn can signal for protein degradation, alter cellular location as well as prevent or promote protein-protein interactions [43–45]. RanGAP1 is a GTPase activator, converting the Ras-related nuclear regulatory protein Ran to its putatively inactive GDP-bound state [46]. Recently, it has been shown via comparative proteomic analysis that RanGAP1 is differentially expressed in diffuse large B-cell lymphoma (DBCL) and that a multikinase inhibitor induces cell death, hyperphosphorylation and mitotic cell arrest of RanGAP1 in DLBCL cell lines but not in normal B and T cells. This suggests a potential biomarker as well as therapeutic target for aggressive B-cell lymphoma [47].

**Fig. 8:**
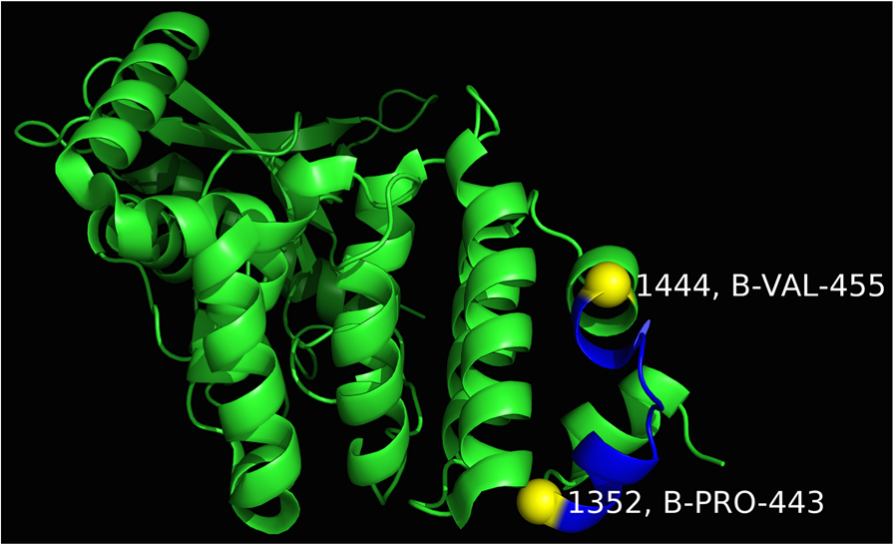
The atoms with serial numbers 1352 and 1444 are labeled and highlighted in yellow. The amino acids between those two atoms are shown in blue

For this structure there was one statistically significant cluster identified in Ran GTPase-activating Protein 1 (UniprotID: P46060) shown in Table 5 and Fig. 8.

**Table 5:** Cluster identified by *GraphPAC* for structure *2GRN*

Cluster	Residues in cluster	Start serial	End serial	Num. Muts	*p*-value
1	13	1352	1444	3	5.39E-03

For the cluster we show: 1) the number of residues in the cluster, 2) the beginning and ending serial number, 3) the number of mutations in the cluster and 4) the *p*-value

It is worth noting that the cluster is nearby amino acid 442 which is phosphorylated at the onset of mitosis and is associated with RanBP2 regardless of its phosphorylation state. As such, the phosphorylation is believed to potentially effect RanGAP1’s catalytic activity or allow RanGAP1 to recruit specific SUMO target proteins to RanBP2’s catalytic domain [48].

### *SpacePAC* identifies new proteins with clustering

Finally, we now consider structure *2YDR* [49], one of the 21 structures identified by *SpacePAC* when considering the entire protein macromolecule. *2YDR* consists of two protein fragments, one of which is tumor antigen P53 (TP53). TP53 is a well known tumor suppressor involved in cell cycle regulation and apoptosis [50, 51] and is responsible for encoding a transcription factor that is activated in response to cellular stress [52]. The majority of TP53 mutations (over 75 %) correspond to missense mutations [53], and approximately 30 % of all TP53 missense mutations occur in CpG dinucleotides [54]. TP53 somatic mutations have been associated with a wide variety of cancers including acute myeloid leukemia [55], colorectal cancer [56] as well as nonsmall cell lung cancer [57]. Moreover, TP53 germ-line mutations have been shown to be the underlying cause of Li-Fraumeni syndrome [58], a rare autosomal dominant hereditary disorder that predisposes the individual to cancer.

**Fig. 9:**
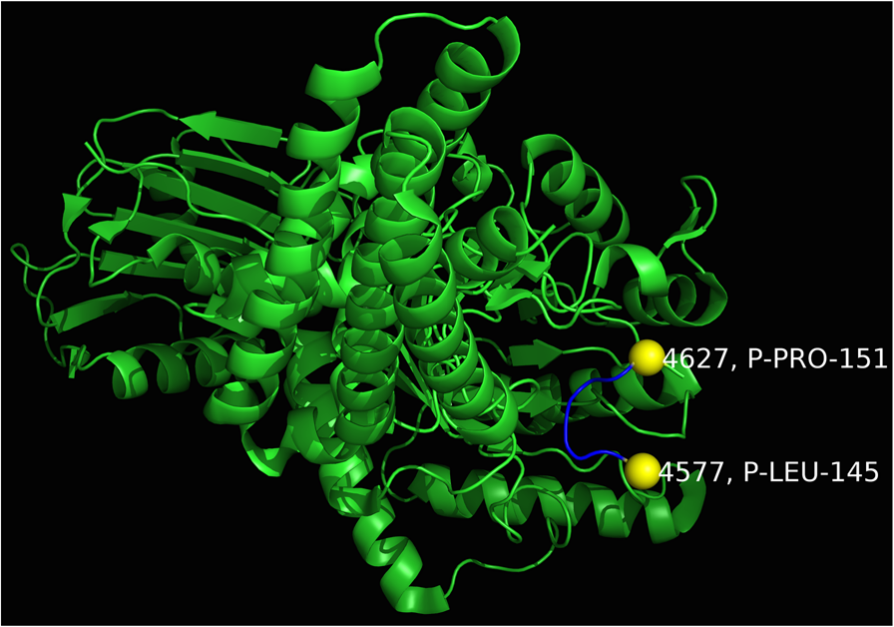
The atoms with serial numbers 4577 and 4627 are labeled and highlighted in yellow. The amino acids between those two atoms are shown in blue

While clusters involving the TP53 protein were found in many of our structures when both the quaternary and tertiary structures were considered, the hotspots shown in Table 6 and Fig. 9 are unique only to the quaternary structure. Not only have mutations in that region occurred in sporadic cancers in the case of Li-Fraumeni syndrome, it is also worth noting that P151S (serial number 4627) is associated with squamous cell carcinomas [59]. It is worth noting that in recent years, significant resources, have been spent to drug the TP53 pathway in order to arrest further tumor growth [60–62].

**Table 6:** Clusters identified by *SpacePAC* for structure *2YDR*

HotSpot	Sphere center serial	Sphere radius (Å)	# Mutations
A	4577	3	3
B	4627	3	5

Both hotspots A & B were identified by *SpacePAC* at an optimal radius of 3Å

## Conclusion

In this manuscript we expand upon several previous methodologies in order to account for protein quaternary structure. By utilizing the entire macromolecule that is comprised of several protein subunits we are able to identify several structures with statistically significant clusters that are otherwise missed. Moreover, we demonstrated several examples where the clusters identified may have a potential therapeutic benefit and in some cases, are already currently being targeted by the pharmaceutical and biotech industries. Furthermore, when considering individual protein subunits, many structures are blank in that they don’t have enough mutations to evaluate whether a cluster exists. As our approach considers the entire protein molecule, it is often able to classify whether or not a cluster occurs (even if all the individual subunits are “blank”) by leveraging mutations over all the subunits within the quaternary structure. This type of negative result can provide valuable insight for the wet-lab scientist when screening many compounds to decide which one requires further evaluation. Finally, although we consider larger structures in this approach, the impact on the running time of *iPAC*, *GraphPAC* and *SpacePAC* is negligible when compared to analyzing the tertiary structure. Most structures are analyzed within 10-15 minutes when the software is run on a consumer desktop with an Intel i7-2600k processor and 16 GB of RAM.

While utilizing the quaternary structure is a significant improvement, this methodology is still subject to some of the same limitations as the tertiary approaches. For example, our approach does not allow for unequal rates of mutagenesis in specific genome regions. To help minimize the impact of this assumption, we considered only missense substitution mutations due to the fact that many insertion and deletion mutations are dependent upon sequence location. Further research is required in order account for other genomic mutational hotspots such as CpG dinucleotides which may have mutational rates that are 10 times higher than other locations [63]. However, as most of the clusters identified are similar when considering the tertiary versus quaternary structures, the impact of such hotspots is limited as described by [24, 26]. Our approach also doesn’t account for differences in mutational position due to the type of mutation. For example, cigarette smokers often result in lung carcinomas with transversion mutations [23] while colorectal carcinoma pathologies often demonstrate transition mutations [64]. However, KRAS mutations, which are often present in both of these carcinomas, nevertheless have the vast majority mutations on residues 12, 13 and 61 for both cancers suggesting that the mutation type may only have a small impact on the uniformity assumption [25]. In all, while this approach may still be influenced by a variety of factors that we are unable to account for, it does suggest that utilizing the quaternary structure is beneficial when identifying statistical clusters.

In summary, *QuartPAC* provides a new (and as far as we are aware, only) tool for researchers to statistically identify mutational clustering when considering the multi-subunit quaternary structure. We show that many of the novel clusters identified have biological and potentially therapeutic relevance. Moreover, by considering the larger oligomeric structure, the additional information provided by the mutations in all the subunits may allow a scientist to definitively rule out a protein structure that would otherwise not have enough data to be classified, providing valuable time savings when many proteins need to be considered. Several promising areas of additional research are self evident such as loosening the requirement that mutations occur uniformly throughout the genome under the null hypothesis. Also, while we present the results here using human missense mutational clusters within proteins, the approach can also be directly applied to both DNA and RNA, as long as the structural data are available.

## Ethics statement

Our work only involved information already published or publicly available via pdb.org and cancer.sanger.ac.uk. No human or animal data was collected. As such, our work did not need to be reviewed by an ethics committee.

## Consent

This article is not a prospective human study nor does it present individual clinical data. All clinically relevant data is referenced to other already published articles.

## Endnote

^1^ Blanks are defined as structures where there is at most one mutation. Thus, by definition, no clustering is possible.

## Additional files

Additional file 1 Cosmic query. Shows the SQL query used to extract mutations from the COSMIC database. (DOCX 82.6 kb)

Additional file 2 Structure files. (XLSX 344 kb)

Additional file 3 Methodology results. A summary of the clustering outcome for each structure broken out by subunit. (XLSX 393 kb)

Additional file 4 Quaternary vs tertiary. Shows the *p*-value along with the number of amino acids (for *iPAC* and *GraphPAC*) or amino acid center location (for *SpacePAC*) for each of the structures deemed significant under each method. (XLSX 61.7 kb)

Additional file 5 Trinary outcomes. A summary for each structure that denotes if there was statistically significant clustering or not when evaluated under tertiary and quaternary methods. Structures that are blank are demarcated as well. (XLSX 96.7 kb)

Additional file 6 Performance evaluation. In-depth analysis of the quaternary methodologies when compared with *PolyPhen-2* and *CHASM*. (XLSX 41.7 kb)

Additional file 7 OMIM classification. A summary of each structure cross-referenced against the OMIM database. Additional statistical measures comparing performance are also shown. (XLSX 343 kb)
