# Supplementary material for: Leveraging protein quaternary structure to identify oncogenic driver mutations
Source: BMC Bioinformatics. 2016 Mar 22;17:137. doi: 10.1186/s12859-016-0963-3 (PMC4802602; doi:10.1186/s12859-016-0963-3)
Supplement: Additional file 1 — Cosmic query. Shows the SQL query used to extract mutations from the COSMIC database. (DOCX 82.6 kb) [file 12859_2016_963_MOESM1_ESM.docx]

CREATE TABLE test AS

SELECT

GSO.gene_name,SA.id_sample,

upper(REPLACE(sa.sample_name, '-','')) as sample_name,gsm.id_mutation, SM.AA_mut_start, SM.AA_MUT_STOP,length(TR.TRANSCRIPT_AA_SEQ) as AA_len,

SM.AA_MUT_SYNTAX,

GSO.swissprot_accession,

SA.ID_SOURCE_SPEC,SA.ID_source,

GSM.ID_MUT_SOMATIC_STATUS,

gsm.ID_MUT_VERIF_STATUS,

SA.ID_SOURCE_TISSUE_ORIGIN,

TR.accession_number, GS.WHOLE_GENE_SCREEN,

GS.WHOLE_GENOME_SCREEN FROM ANALYSED_GENE_SAMPLE T, GENE_STUDY GS,

Gene_sample_mutation GSM,

sequence_mutation SM ,

cosmic_tumour_sample_overview SA,

gene_study_transcript GST,

transcript TR,

Gene_SOM GSO

WHERE

GS.id_gene_study = T.id_gene_study AND

GSM.id_ags=T.ID_AGS AND

SM.id_mutation=GSM.id_mutation AND

SA.id_sample=T.id_sample AND

GST.id_gene_study=GS.id_gene_study AND

TR.id_transcript = GST.ID_TRANSCRIPT AND

GSO.id_gene=TR.id_gene AND

GSM.ID_MUT_SOMATIC_STATUS in (1, 2) AND GSM.ID_MUT_VERIF_STATUS=50 AND

SM.id_mut_type_AA=22 AND

(GS.WHOLE_GENE_SCREEN='y' OR

GS.WHOLE_GENOME_SCREEN='y' OR

sa.sample_name like 'TCGA%' OR

sa.sample_name like 'ICGC%' OR

sa.sample_name like 'CGP%' OR

sa.sample_name like 'LUAD%' OR

sa.sample_name like 'LC_%')

ORDER BY

gs.id_gene,SM.AA_mut_start,sa.sample_name;
